# Efficient and secure outsourcing of genomic data storage

**DOI:** 10.1186/s12920-017-0275-0

**Published:** 2017-07-26

**Authors:** João Sá Sousa, Cédric Lefebvre, Zhicong Huang, Jean Louis Raisaro, Carlos Aguilar-Melchor, Marc-Olivier Killijian, Jean-Pierre Hubaux

**Affiliations:** 1 Laboratory for Communications and Applications - LCA 1, École Polytechnique Fédérale de Lausanne, Route Cantonale, Lausanne, 1015 Switzerland; 2 Laboratory for Analysis and Architecture of Systems - LAAS-CNRS, Université Toulouse, 7 Avenue du Colonel Roche, Toulouse, 31400 France; 3 Toulouse Institute of Computer Science Research - IRIT, Université Toulouse, 118 Route de Narbonne, Toulouse, F-31062 France

**Keywords:** Secure outsourcing, Homomorphic encryption, Private information retrieval, iDash, Genomic variants

## Abstract

**Background:**

Cloud computing is becoming the preferred solution for efficiently dealing with the increasing amount of genomic data. Yet, outsourcing storage and processing sensitive information, such as genomic data, comes with important concerns related to privacy and security. This calls for new sophisticated techniques that ensure data protection from untrusted cloud providers and that still enable researchers to obtain useful information.

**Methods:**

We present a novel privacy-preserving algorithm for fully outsourcing the storage of large genomic data files to a public cloud and enabling researchers to efficiently search for variants of interest. In order to protect data and query confidentiality from possible leakage, our solution exploits optimal encoding for genomic variants and combines it with homomorphic encryption and private information retrieval. Our proposed algorithm is implemented in C++ and was evaluated on real data as part of the 2016 iDash Genome Privacy-Protection Challenge.

**Results:**

Results show that our solution outperforms the state-of-the-art solutions and enables researchers to search over millions of encrypted variants in a few seconds.

**Conclusions:**

As opposed to prior beliefs that sophisticated privacy-enhancing technologies (PETs) are unpractical for real operational settings, our solution demonstrates that, in the case of genomic data, PETs are very efficient enablers.

## Background

Due to the impressive advances in high-throughput technologies, we have witnessed a significant deluge, in the last few years, of digitalized genomes. This increasing availability of genomic information has triggered massive research in the so-called *“data-driven medicine”*, thus paving the way to the new era of personalized health. Yet, even if the promise of personalized diagnoses and treatments seems just around the corner, the required storage and processing capacities necessary to run analyses on these data are becoming increasingly prohibitive and often beyond the capabilities of single institutions. For this reason, many medical research centers and healthcare providers are beginning to look into cloud computing [1] as a flexible and cost-effective solution to outsource the expensive storage and processing of genomic data.

Pushing genomic data to a cloud, however, is not an easy task. One of the main difficulties stems from the important privacy and security concerns caused by outsourcing these sensitive and personal data to an untrusted third party. Indeed, unlike other types of medical data, genomes cannot be anonymized [2, 3]. Many recent attacks based on either side-channel information [4], phenotype/genotype correlations [5], or genealogical triangulation [6] have shown that standard anonymization techniques are ineffective with genomic data and, as a consequence, de-identified genomes can be easily re-identified. Furthermore, although the recent NIH (National Institutes of Health) Genomic Data Sharing Policy [7] permits NIH-funded studies to use public cloud infrastructures to facilitate large-scale data analyses, it also states that the data owner (i.e., a researcher or an institution), instead of the cloud provider, is responsible for data security and privacy. In other words, if a data breach occurs, the accountable party is the one that stores and processes the genomic data on the cloud and not the cloud service-provider. The leakage of genomic information can open the door to all sorts of abuse and threats, not only for the individual but also for his relatives. Hence, effective protection mechanisms have to be put in place to protect genomic data when their storage and processing are outsourced to an untrusted cloud environment.

In response to these concerns, over the last few years, the privacy and security community has proposed several techniques for securely outsourcing the storage and processing of genomic data to cloud environments. The most popular ones are those based on homomorphic encryption (HE), the state-of-the-art cryptographic technique that enables a party to perform certain computations directly on the encrypted data and decrypt only the final result, thus preserving the confidentiality of the raw genomic sequences from an honest-but-curious cloud provider. For example, McLaren et al. [8] propose a technique, based on additive HE, for securely performing pharmacogenetic tests on encrypted genomes. Similarly, Lauter et al. [9] show how several statistical algorithms can be carried out on encrypted genomes by using somewhat HE (SHE). Naehrig et al. [10] also show how certain approximated machine-learning algorithms can be securely run in the cloud by using HE. Finally, Wang et al. [11] make use of HE to securely compute exact logistic regression.

However, most of these solutions have had limited adoption because of either (i) their lack of flexibility (i.e., some privacy-preserving solutions can be used only for specific tasks on specific types of data such as SNPs) or (ii) their cost (HE introduces a significant storage and computational overhead that substantially impairs scaling to real-size genomic datasets).

In this paper, we address this problem by proposing a novel and very efficient solution for securely outsourcing genomic-data storage and processing that outperforms the state of the art. Our solution is based on HE and private information retrieval (PIR) [12] and enables a user to securely store millions of genomic variants of all types for one or multiple individuals on the cloud and to efficiently search for specific genomic variants without revealing anything to the cloud provider.

Due to its efficiency, the proposed solution was selected among the finalists at the 2016 iDash competition [13], which was held in Chicago, Illinois, USA on November 11, 2016. The iDash competition is a community-wide open competition whose goal is to bridge the gap between the biomedical informatics, data privacy, and security communities by benchmarking new secure solutions for known genomic-security problems. The intention is to address these issues and further advance the current state of the art in the genome privacy and security research field.

We summarize the key contributions of this paper:

- A new secure and efficient solution to storing and searching genomic data in a public cloud that provides data and query confidentiality and hides access patterns from the cloud.
- The first application of private information retrieval for genomic data.
- A thorough performance evaluation on real genomic data.
- A detailed study of the security/privacy vs. performance trade-offs.

## Methods

Our main objective in this paper is to propose an efficient solution for securely storing and searching genomic variants in a public cloud. The cloud must not find out any information associated with the data kept within its premises but must still enable the data owner the ability to query it. These genomic data are kept in variant call format (VCF) files, one for each individual, under the following structure:

$$\begin{array}{@{}rcl@{}} \begin{array}{c} \textsc{chromosome} \| \textsc{position} \| \textsc{variant id} \| \textsc{reference} \|\\ \textsc{alternate} \| \ldots \hphantom{a} \end{array} \end{array} $$

**Fig. 1:**
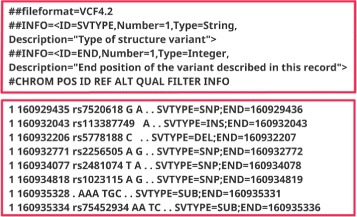
Variant Call Format (VCF) file. This is a text file that stores genomic information, in particular, genetic variations, for example, single-nucleotide polymorphisms (SNPs). Each VCF file is divided into a *header* section, which provides some meta-data describing the remaining content of the file, and the *body*, which contains all the different variants

The reference and alternate alleles represent, respectively, the alleles or set of nucleotides that normally are present, and their substitutes. All other fields are self-explanatory. It is important to mention that the variant id is sometimes absent. Figure 1 provides an example of one of these files and some of their content. To correctly query for a specific variant, we need to specify only four parameters: chromosome, position (in the chromosome), reference and alternate. The first two parameters provide the location of the variant in the genome, and the other two represent the associated allele mutation that can be different from individual to individual.

For the remainder of this paper, we assume symmetric non-homomorphic encryption under a key *K* (e.g., AES) to be represented as *E*_*K*_(·) and homomorphic encryption as *H* *E*_*K*_(·). Hashing is symbolically represented as *h*(·).

### Background

Our solution draws inspiration from a popular privacy technique known as private information retrieval. This protocol is of particular interest in a scenario when one of the participating parties (e.g., a server) owns a database and another party wants to perform one or more queries without leaking any information, such as access patterns. Conceptually speaking, we want to achieve the same level of privacy as we have when downloading the entire database from the server and performing the search locally. There are many variations of this protocol, but the current model for our solution uses *cPIR* [14] (Computationally Private Information Retrieval) with HE, which safeguards against a polynomially-bounded querier. The way this protocol works, in practice, is to make each query indistinguishable and to ensure that the server processes all of its database entries. In this way, it cannot obtain any information, either by looking at the query or by looking at the computations performed on the database.

### System model

**Fig. 2:**
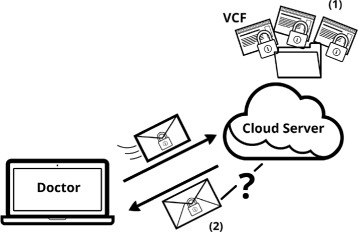
Doctor-Server setting: *(1)* an honest doctor securely stores his patients’ genomic data in a cloud service; *(2)* he performs search queries on that data without compromising any of his patients’ confidentiality and privacy

Our system comprises a data owner/client who possesses the genomic information in multiple VCF files. This information is sent to a cloud server to be stored in a database, as depicted in Fig. 2. The client can then query the server to find out if one or more variants are present in the data bank. This information can be used for multiple purposes and is particularly important for genome-wide association studies (GWAS) that statistically assess the correlation between genetic variants and disease status. Our solution is able to hide data, query and access patterns from the cloud.

We envision simple scenarios such as a doctor who possesses the genomic data of his patients and wants to consult this information, or a patient who stores his genomic information on an external cloud service to free some space in his device.

### Threat model

Our system is concerned mainly with the confidentiality of data, such as genetic variants and with side-information such as access patterns. We assume an honest-but-curious cloud server that follows the defined protocol but might try to infer, during its execution, sensitive information from the owner’s data. This model is useful in cloud computing, because a cloud’s malicious behavior, such as tampering with the computation results, can be caught with a periodic system audit, e.g., where the owner downloads a random small subset of the data and verifies previous computation results [15]. The server can be instantiated with well-known cloud service providers (e.g., Google, Amazon, Microsoft) that have business incentives to behave honestly and are prudent to avoid malicious behaviors. We do not consider any malicious adversaries on the communication channel, as it is authenticated, confidential, and integrity-protected with state-of-the-art security techniques (e.g., TLS 1.2 [16]). The client, who is the data owner and is always authenticated, is assumed to be honest.

### Proposed solution

With the previous system model in mind, we devised a hash-based solution using homomorphic encryption and PIR. We made some changes to the standard PIR protocol in order for it to have access to a given variant using its identification parameters (chromosome, position, reference allele, alternate allele), rather than its relative position in the VCF file. Furthermore, we protected the genomic data at the server side by means of symmetric encryption.

We decided to split our solution into *initialization* (encoding and encryption) and *querying* phases, in order to separate between one-time offline operations (e.g., hashing, encrypting and uploading data) and online interactive operations that need to be executed each time a request is performed (e.g., generating query, obtaining the response).

#### Initialization phase

The **initialization phase** (Fig. 3) comprises the following steps:

1. The client (e.g., doctor) generates a symmetric key, *S*, to later protect the sensitive data by using symmetric encryption. He also generates another key, *U*, to compute hashes and a pair of public, private keys, (*R*,*r*), for the homomorphic encryption scheme.

**Fig. 3:**
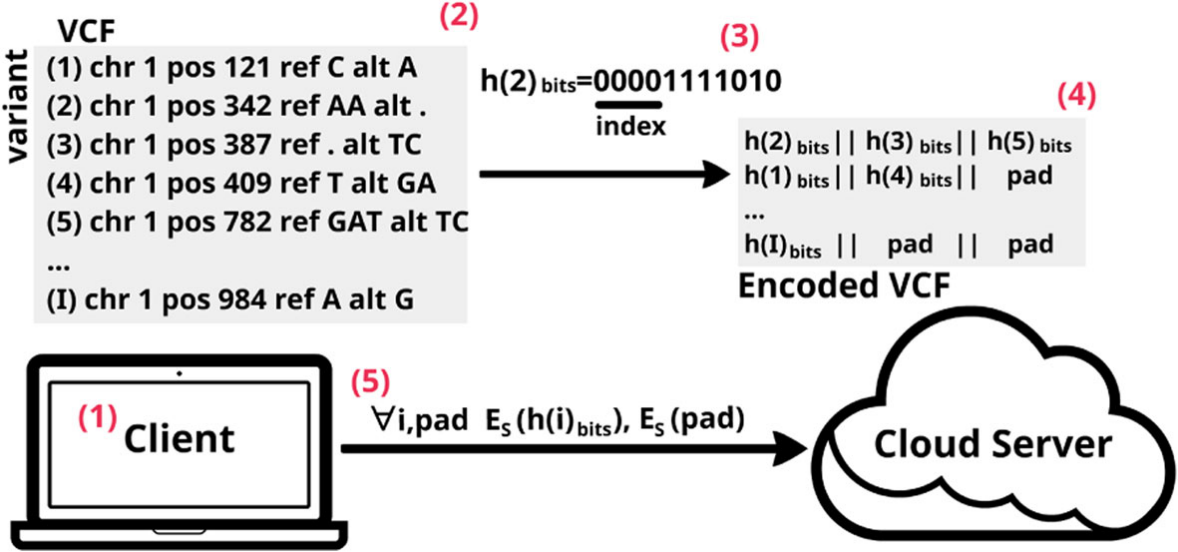
Initialization phase. The owner of the data first encodes the data (variants 1 …I) into a new VCF file, which is then symmetrically encrypted and sent to the cloud server
2. The client possesses multiple VCF files in his machine that list the genomic variants {1…*I*}, where *I* is the total number of variants. For example, in case of a medical doctor, each VCF would contain the variants pertaining to a specific patient. He computes for each variant *i* a hash, *h*(*i*), using *U* and keeps the first data_hash_size bits, *h*(*i*)_*bits*_. Hence, data_hash_size quantifies the size of the hash representation that is going to be stored in the server. A smaller data_hash_size would greatly improve performance, but would increase the probability of collisions, or in other words that two variants are represented by the same hash.
3. From *h*(*i*)_*bits*_ he extracts x bits that will map to an index. The hash, *h*(*i*)_*bits*_, is then stored in another file (encoded VCF) in this new position. A larger x means that the server’s database will have more entries (2^*x*^) since the number of possible mapped indexes is bigger. A smaller x reduces the number of possible indexes and, as a consequence, increases the number of collisions in the new encoded VCF file. If a collision occurs, the variant is concatenated to pre-existent ones.
4. All rows in the encoded VCF file, even if empty, are padded so that they have the same length.
5. Finally, each element in a row is symmetrically encrypted. The combination of padding and symmetric encryption ensures that all entries have the same size and are indistinguishable.

#### Querying phase

The **querying phase** (Fig. 4) is mostly the same as standard PIR, repeated for each variant and/or VCF file queried:

1. The client specifies the query and calculates the hash for the variant *j* being searched using the same key, *U*, as before, *h*(*j*)_*bits*_. He then maps it to its respective index, pos, using the first x bits of that hash.

**Fig. 4:**
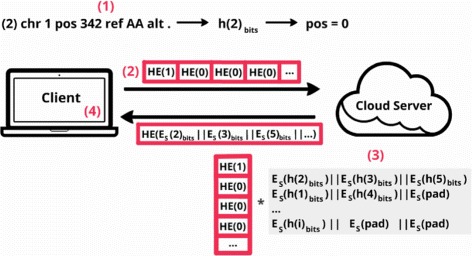
Querying phase. The owner of the data first maps the queried variant into a database index and then runs a generic homomorphic version of the PIR protocol
2. The client issues a PIR query for that position. In other words, the client sends an array, arr, with the same length as the queried VCF file in the database, composed of homomorphic encrypted 0s and a 1 on the position of the desired element
  $$\rightarrow~\forall l \in \overline{arr}: \left\{\begin{array}{cc} HE_{R}(1) & \quad \text{if} \;l = \text{pos}\\ HE_{R}(0) & \quad \text{otherwise}\\ \end{array}\right. $$
  Note that the encryption scheme is probabilistic (there are many encryptions of 0 and of a single 1) and that the encryptions of 0 and 1 are indistinguishable for an attacker.
3. The server generates a PIR reply. To do that the server multiplies each value in the array by its respective element in the queried VCF file and adds up everything. When there is a 1, that entry is homomorphically absorbed (i.e., *H* *E*(1)×*y*=*H* *E*(*y*)), otherwise it is erased. As encryptions of 0 and 1 are indistinguishable, the server cannot know which entry is absorbed and which ones are erased. If the variant exists it will correspond to one of the elements/variants in the response vector.
4. Finally, the response is sent to the client, who decrypts it, using *r* and *S*, and checks for the presence of the variant.

**Fig. 5:**
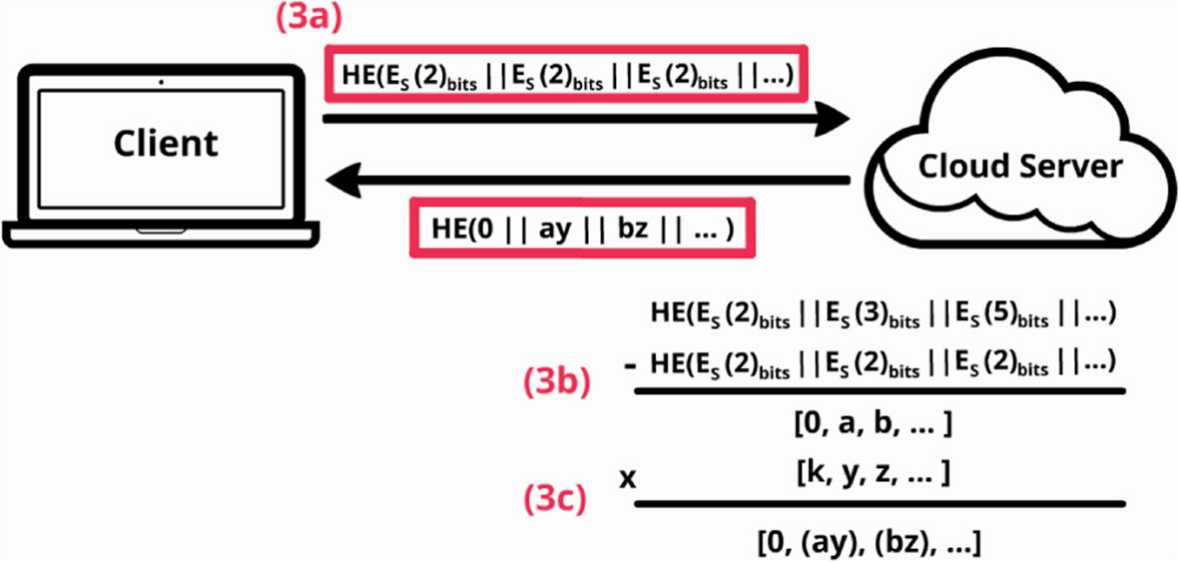
Subtraction step. The server subtracts the encrypted hash of the variant with its PIR reply and then randomises the result, thus hiding all other variants except for the one being queried
- The server then subtracts its PIR reply with sub. If the variant is present in the reply vector some of the polynomial coordinates will be 0.
- Finally, the server multiplies each element of the reply vector by a multiplicative mask. This way, it randomizes every element in the response by using an uniformly random multiplier (one-time pad), except the zero coordinates that exist only if the reply contains the variant. Hence, the client only has to search for consecutive zero polynomial coordinates after decrypting the result to check whether the variant is in the file or not. Yet he cannot obtain any other information as non-zero entries have been randomized.

In this solution, the client queries a single row where he thinks a specific variant is. However, as each row contains multiple elements, he will have access to extra information, aside from the variant that he is looking for. Most of the time, these additional data do not raise any security or privacy concerns as they belong to the client himself. However, if we consider an honest-but-curious querier (e.g., an outside entity, such as a medical researcher, who is authorised to query the database), retrieving more information than intended can pose privacy problems. Therefore, we propose an extension to our *querying phase* to be performed after the PIR reply-generation denoted **subtraction step**, depicted in Fig. 5:

- In addition to the PIR query, the client sends a “subtract query”, sub, which contains the symmetrically encrypted hash of the variant being searched, *E*_*S*_(*h*(*j*)_*bits*_), replicated multiple times so that it has the same length as a row in the queried file.

### Implementation

In our solution, we make use of a very recently improved private information retrieval implementation, *XPIR* [17], combined with AES_CTR256 for symmetric encryption and HMAC_SHA256 for hashing. The XPIR library has been modified to include an optimized Fan and Vercauteren (FV) [18] homomorphic encryption scheme.

There are a number of reasons for choosing these particular techniques. First, for the protection of data at rest, we opted for AES_CTR256 because it is considered safe for today’s systems, but it also dramatically reduces the storage cost at the server side. In fact, the size of each ciphertext is the same as the corresponding plaintext value and the data owner can simply store locally the 64-bit nonce needed to generate the initialization vectors (IVs). Each IV corresponds to this random nonce, different for each encoded VCF file, concatenated with each variant’s position (64 bits). Second, for hashing, HMAC_SHA256 provides a collision-resistant function proven to still be secure. Finally, for homomorphic encryption, we use FV-NFLlib [19] that is an implementation of FV based on NFLlib developed for the HEAT [20] project. NFLlib is an efficient library dedicated to ideal lattice cryptography and is currently employed by the XPIR application due to its security guarantees (i.e., uses secure Gaussian noise generators).

For padding, various schemes could be applied in order to distinguish dummy elements from real ones. We refer to the PKCS #7 padding scheme [21], where the value of each dummy element is equal to the number of dummy elements. For example, if we have to pad 3 dummy elements, the padding will be “ 3∥3∥3”.

### Parametrization

For the keys of HMAC and AES, we chose sizes that are considered to be standard and secure. The remaining parameters had to be empirically fine-tuned. Table 1 contains the different parameters of our system.

**Table 1:** List of parameters

Parameters	Description
DATA_HASH_SIZE	Length in bits of a variant’s hash to be stored.
BITS FOR MAPPING = *x*	Number of bits extracted from the hash that maps to a specific index.
NUM_ENTRIES	Number of entries in the encoded VCF (=2^*x*^).
ROW_SIZE	Number of elements per row of *d* *a* *t* *a*_*h* *a* *s* *h*_*s* *i* *z* *e* bits. Indirectly defines the number of dummy elements (padding) to be added.
ENCRYPTION MODE	Cryptographic parameters for the FV scheme: FV:A:B:C:D. FV is to be used with A security bits, polynomials of degree B, polynomial coefficients of C bits and capable of absorbing a maximum of D bits per coefficient.
AGGREGATION	Number of aggregated rows. Ensures that multiple rows are concatenated resulting in a database with a lesser amount of rows (NUM_ENTRIES/AGGREGATION), which are in turn longer (ROW_SIZE × AGGREGATION).
DIMENSIONALITY	Level of recursion.

We first analyze the *d* *a* *t* *a*_*h* *a* *s* *h*_*s* *i* *z* *e* parameter. For this particular variable, more bits will reduce the chance of having false positives (one variant with the exact same hash as another one), but will also increase the overall database size. For example, storing 48 bits of each variants’ hash in a five million VCF file would lead to a false positive probability of roughly $5,000,000/2^{48} \approx \frac {1}{2^{25}}$. We consider this to be acceptable, as the error rate in DNA sequencing is well above this probability [22]. Nevertheless, we could increase the stored hashes to 96 bits so as to have a cryptographically low probability of false positives, thus doubling the database size, as well as the *subtract query* size, and multiplying the response time by slightly less than two (as the PIR query size is unchanged).

Varying the number of bits for mapping *x* affects multiple dimensions of our solution. The entries of each encoded VCF are indexed by the *x* first bits of the hash of each variant. Hence, each entry is a list of encrypted variants that map to the same index i.e., the first bits of their hash are the same. Choosing a small *x* significantly increases the average number of collisions and reduces the number of entries, *n* *u* *m*_*e* *n* *t* *r* *i* *e* *s*. Subsequently, the number of collisions defines the amount of padding needed, or in other words the *r* *o* *w*_*s* *i* *z* *e*. For example, if the maximum number of collisions in one single index, among the different VCF files, is 100, then we can set our *r* *o* *w*_*s* *i* *z* *e* to the same number and, with that, homogenize the size of each VCF file in the database.

On the contrary, a higher *x* means a lengthier encoded VCF file, as more bits are extracted during our mapping phase. This reduces the number of collisions but significantly increases the amount of dummy data needed to hide every entry.

In practice, if we have *x*=13, then we have *n* *u* *m*_*e* *n* *t* *r* *i* *e* *s*=2^13^ elements in each encoded VCF file. As a result of the *law of large numbers* [23], the average maximum number of collisions would be around 710 for a five million variants’ file. As such, the padded VCF size will be reduced by a factor between 4 and 6, compared with another VCF file with 2^22^ elements with an average of 1 collision, but with the maximum number close to 5–6. Such an improvement will reduce pre-processing by the same factor, as well as, by roughly a factor 2, query-generation/query-sending and reply-generation times. Nevertheless, there is a limit to how compact each file can be, as the increasing number of collisions greatly expands the size of the reply.

Both *aggregation* and *dimensionality* are associated with the PIR scheme. The first parameter enables the *packing* of data, that is, enables multiple rows to be concatenated into a single row. Thus, during the PIR protocol, we can reduce the number of rows of a file in the database, as well as the size of PIR query, in exchange for a bigger PIR reply.

**Fig. 6:**
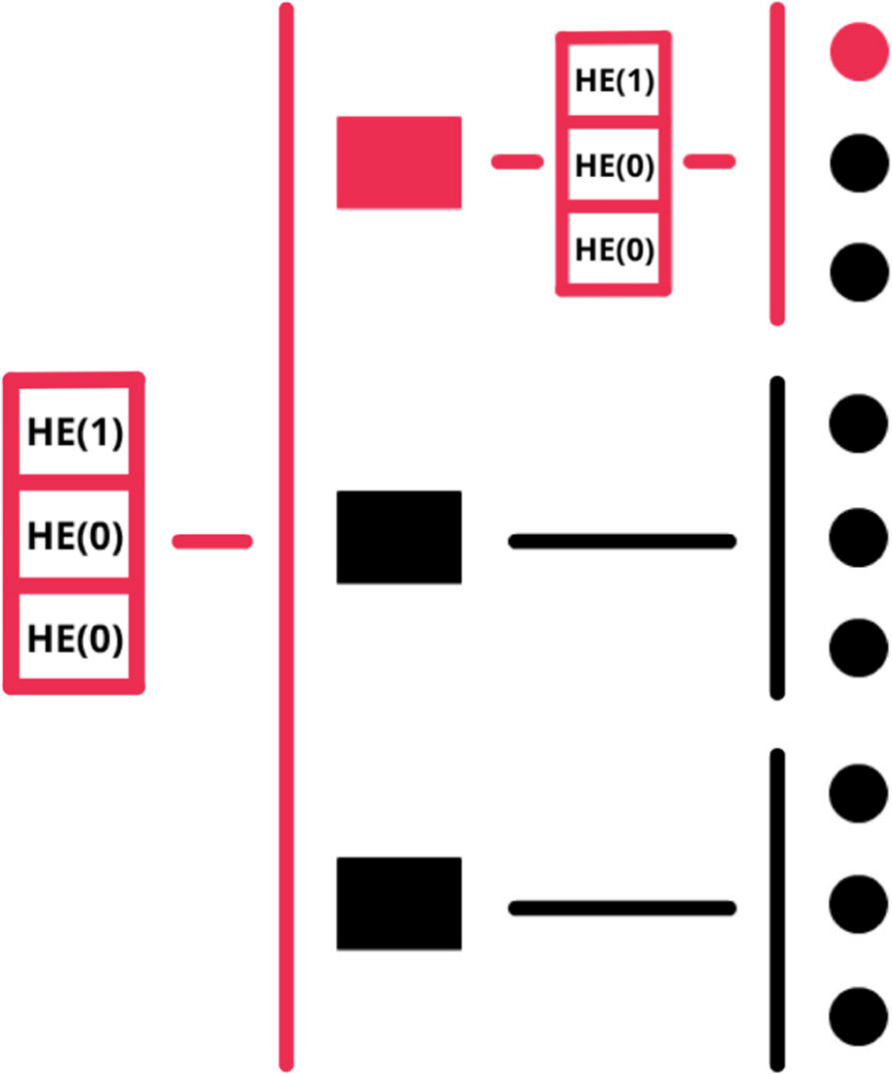
Dimensionality/Recursion. With *d* *i* *m* *e* *n* *s* *i* *o* *n* *a* *l* *i* *t* *y*=2 the size of the PIR query is smaller but the server needs to perform more iterations to generate the correspondent reply. In the example above, we reduce the size of the PIR query from 9 to 6 elements

The second parameter, *dimensionality*, enables the recursive execution of multiple PIR queries simultaneously (Fig. 6), thus reducing query transmission time. However, the reply size grows exponentially in the number of dimensions, hence we must keep the dimension small (<4). Both dimensionality and aggregation can be chosen in a way that ensures maximum performance.

### Security analysis

Our solution uses multiple techniques, in particular, two encryption mechanisms, a hash and a padding scheme that, when combined, provide data and query confidentiality and hide the access patterns from the cloud server.

The first encryption scheme, AES-CTR256, outputs a uniformly random distributed ciphertext for each encrypted variant. Therefore, the data sent to the cloud cannot be directly used by an attacker to obtain confidential information.

The second encryption scheme, FV, is a somewhat homomorphic encryption (SHE) scheme that ensures indistinguishably against chosen plaintext attacks and is limited to an amount of operations (e.g., some additions and/or multiplications). Beyond this limit, an operation on the ciphertext creates too much noise for the decryption to be correct, as the noise overflows the data. FV is based on the ring-learning-with-errors (RLWE) problem; and to estimate the security of its parameters we use Martin Albrecht’s work [24] that provides a script to generate this information based on existing attacks. The script returns the security of the most recent attacks against LWE-based cryptosystems, and we assume the results hold for RLWE. This is the standard approach to estimating RLWE security nowadays.

Hashing, SHA256, ensures that we can represent any kind of variant (SNPs, insertions, deletions, etc.) in a compact way and still provide indistinguishability, after we symmetrically encrypt the data. By padding, we ensure that each row in each of the files in the server is the same size, hence the line length does not leak any information.

Finally, the *subtraction step* changes the solution so that the querier can learn only about the requested variants and nothing else.

By combining all of these techniques, we ensure data confidentiality and privacy throughout the two main steps of our solution. For the first step, the initialization phase, symmetrically encrypting the hash of the variants using the client’s private key, ensures that no one besides the client can decrypt this information. As for the second step, the querying phase, as the query has the same length as the length of the queried file and all its elements are homomorphically encrypted, the server cannot obtain any information about the rows that the client wants to access. In addition, the server cannot distinguish between two different queries, as the encryption scheme used to generate them provides ciphertext indistinguishability based on a standard cryptographic assumption. The operations performed at the server side (to obtain the PIR reply) are always done between homomorphic-encrypted data and symmetric-encrypted data, therefore the stored variants and the query are protected at all times. The server performs the same homomorphic multiplication for every single row of the VCF file thus does not know which one is being retrieved.

The extension to our solution simply reduces the amount of extra data that the client has access to. Data are still encrypted before, during, and after the subtraction step, hence there is no leakage of information.

## Results

In this section, we assess, in different settings, the performance of our system and offer a comprehensive view on how to achieve better results. We split our results in two categories: a generic performance evaluation and a specific evaluation focused on the tests conducted during the iDash Challenge.

We ran both client and server, implemented in C++, on a Ubuntu (64-bit) virtual machine with 4 GB RAM and 250 GB hard disk, on top of a MacBook Pro with a 3.1 GHz Intel Dual-Core i7 processor. We enforced a 10 Mbps link for both downloading and uploading data. Each measurement was averaged over 10 independent runs and we displayed the standard deviation for the round-trip time (RTT).

### Generic performance evaluation

Table 2 lists five different settings, and Table 3 showcases the results. All different setups will **run a single variant search on a specific VCF file with five million variants**. We generated this test file by using the two datasets provided during the iDASH genome privacy challenge. For more information on each of the parameters, please refer to *subsection - Parametrization*.

**Table 2:** System settings for the generic performance evaluation

Setup	Default	Sparse	no_info
DATA_HASH_SIZE	48	48	48
BITS FOR MAPPING	13	16	13
NUM_ENTRIES	8192	65536	8192
ROW_SIZE	716	130	716
ENCRYPTION MODE	80:1024:62:14	-	-
AGGREGATION	3	15	3
DIMENSIONALITY	2 (53 ×52)	2 (67 ×66)	2 (53 ×52)
Setup	Security		
DATA_HASH_SIZE	48		
BITS FOR MAPPING	13		
NUM_ENTRIES	8192		
ROW_SIZE	716		
ENCRYPTION MODE	172:2048:62:14		
AGGREGATION	3		
DIMENSIONALITY	2 (53x52)		

Each setup pertains to a single scenario →*default*: optimized setup; *sparse*: sparser database (more entries); *no_info*: no extra variants retrieved; *security*: stronger security guarantees. ’-’ means the same value has the previous setting

**Table 3:** Quantitative results for the generic performance evaluation

Setup	Default	Sparse	no_info
Data preparation (s)	19.2	21	19.6
Size of VCF file (Mbytes)	35	51	35
Importation (s)	0.6	1.08	0.71
PIR query generation (s)	0.013	0.017	0.011
Sending query (s)	1.37	1.74	1.49
PIR reply generation (s)	0.38	0.55	0.46
Sending reply (s)	1.03	1.04	1.26
Reply extraction (s)	0.4	0.58	0.49
**Round-trip-time** (s)	**2.4** ±**.006**	**2.8** ±**.002**	**2.7** ±**.001**
Setup	Security		
Data preparation (s)	21.3		
Size of VCF file (Mbytes)	35		
Importation (s)	0.8		
PIR query generation (s)	0.025		
Sending query (s)	2.88		
PIR reply generation (s)	0.56		
Sending reply (s)	1.39		
Reply extraction (s)	0.34		
**Round-trip-time (RTT)** (s)	**4.3** ±**.02**		

The table is divided into three sections: initialization phase, querying phase and overall querying performance. The results are for one single variant search on a specific VCF file with five million variants. The data in boldface represents the round trip time, which provides an overall measure of the efficiency of our solution in the different setups

We evaluate four setups, of which Setup *default* uses the default settings for our strategy. We fine-tuned these parameters to offer the best possible performance.

For storage, we decided to represent each variant with 48 bits of its hash, ensuring a small file size and a negligible probability of error. Furthermore, we also decided in favor of a compact database that enables us to reduce storage complexity and improve PIR query-generation/query-sending time. We ended with 8192 entries per file (*x*=13), as further aggregating the database would make the PIR reply-generation/reply-sending too cumbersome. For the encryption mode and complementary parameters, we chose those provided by the XPIR optimizer - FV:80:1024:62:14 with aggregation 3 and dimension 2. To obtain these values, the XPIR optimizer empirically tests, for a specific file, different combinations of parameters and chooses those that provide the better overall PIR performance and guarantee at least 80-bits security. We opted to include a pipeline execution and pre-importation. Consequently, both query and reply are sent as they are created, and some data are stored in RAM to enable a much faster computation of all PIR operations. It is worth mentioning that the pre-importation is not viable if the number of patients is too high, roughly 100 for this machine, as there is not enough space in the RAM. Finally, to provide data confidentiality and privacy, we selected a 256-bit key for AES encryption, a secure standard, and a *r* *o* *w*_*s* *i* *z* *e* of 716. This is sufficient for hiding the average maximum amount of collisions on any given VCF file up to five million variants, without having too much storage and time overhead and still keeping the size of each entry at the minimum possible.

The three remaining setups enable us to compare the default strategy (compact database) with an alternative one (sparse database), to evaluate the performance of our *subtraction step* and to assess the security scalability of our solution. For Setup *sparse*, we decided on a lengthier database, an alternative solution, with fewer collisions and a higher recursion to mitigate the effect of having more entries. Setup *no_info* removes the extra information retrieved for each query by adding our *subtraction step*. Finally, Setup *security* is similar to the previous one but, this time, the ciphertext size chosen is larger to provide much stronger security (>128 bits).

We evaluate each setup based on three performance parameters (Table 3) in decreasing order of importance: performance, storage complexity and communication cost. The first metric is assessed by looking at the round-trip time (RTT), the PIR query and reply generation, as well as the reply extraction. Both data preparation and importation have a low impact in the overall complexity, as they are executed only once. The size of the VCF file measures the performance in terms of storage, and send-query and send-reply measures the performance in terms of communication cost. Although not indicated in Table 3, sending the VCF file takes approximately *size_of_vcf_file*(MBytes)/1.25 s with a 10 Mbps of bandwidth.

The first setup, Setup *default*, shows the overall best performance of our basic solution under optimized settings. On average, a client can query the system in less than 2.5 s, while protecting the data at rest, the query, and the response from the cloud server. It is very important to mention again that we are using a five million VCF file, currently considered to be an upper-bound to the the maximum number of variants a human can have in his/her genome [25]. For smaller files, the results are exactly the same, except for the amount of time it takes to prepare the data during the initialization phase. Hence, as long as the VCF has less than five million variants, this solution is file independent due to the padding scheme. In other words, querying a VCF file with one variant takes the same amount of time as querying a file with five million variants.

In the second setup, Setup *sparse*, we notice a slight increase in the overall RTT time, mostly due to the fact that we have a higher number of entries, thus causing the PIR query to be larger and more cumbersome. Aggregation can help, up to a certain point, to mitigate this problem, by having multiple entries in one single polynomial/ciphertext. Without it, our PIR query becomes slightly larger; and the time it takes to generate and send it becomes rapidly impractical.

In the third setup, Setup *no_info*, we introduce the *subtraction step* and, as expected, the RTT increases with respect to our default setting. A part of the overhead is caused by generating and sending the extra polynomial and by performing the corresponding homomorphic subtraction and multiplication. The remaining overhead is caused by having to reduce the number of bits that hold the data (the rest is noise) for each coefficient. Only then can we perform the multiplication without having the noise bits overlap the data bits and still provide enough randomness to prevent the client from inferring the extra information.

Finally, Setup *security* simply proves that scaling security is easily achieved by using our secure searching method.

### The iDash Challenge

In the iDash Challenge [13] there were a number of requirements to consider. Accepted solutions had to (1) hide the data, query and access patterns from the cloud server, (2) employ homomorphic encryption, and (3) retrieve/reveal fewer than 20 variants to the data owner during each single variant search. However, for this competition it was not mandatory to hide the number of variants in each VCF file. Therefore, we decided to slightly change the padding scheme and significantly reduce query runtime. In this case, we add only dummy data to homogenize all rows inside a specific VCF. This padding is weaker than our previous one because the server can still distinguish different files by looking at their sizes. Therefore, the cloud knows if a VCF has more or less variants than another one in the database and can consequently infer an approximation of the total number of variants. Note that in the iDash Challenge only SNPs needed to be considered. However, in our solution, we can test for the presence of any mutation (e.g., an insertion of 1000 nucleotides) due to our hash-based representation. Other approaches not using hash-based representations would incur a much higher complexity to enable handling multi-nucleotide mutations with respect to handling SNPs.

Table 4 lists the different settings, and Table 5 showcases the results for the three queries performed by the challenge organisers: (1) query 4 variants in one single VCF file with 10,000 variants; (2) query 4 variants in one single VCF file with 100,000 variants; and (3) query the same variant in 50 different VCF files with 100,000 variants. We opted for two different scenarios, *a.* and *b.*: they map to two different querying methods: scenario *a.* uses the simpler version of our protocol and was the one we submitted to the competition, and scenario *b.* includes our extension, the *subtraction step*, to eliminate any extra information obtained by the querier.

**Table 4:** System settings for the iDash Challenge

Query	1a	2a	3a
DATA_HASH_SIZE	48	48	48
BITS FOR MAPPING	13	17	17
NUM_ENTRIES	8192	131072	-
ROW_SIZE	6	6	6
ENCRYPTION MODE	80:1024:62:14	-	-
AGGREGATION	4	4	4
DIMENSIONALITY	2 (46*45)	3 (32*32*32)	-
Query	1b	2b	3b
DATA_HASH_SIZE	48	48	48
BITS FOR MAPPING	4	9	9
NUM_ENTRIES	16	512	512
ROW_SIZE	672	256	256
ENCRYPTION MODE	80:1024:62:14	-	-
AGGREGATION	2	2	2
DIMENSIONALITY	1 (8)	2 (16 ×16)	2 (16 ×16)

Each setup pertains to a single query scenario →*1*: “query 4 variants in one single VCF file with 10,000 variants”; *2*: “query 4 variants in one single VCF file with 100,000 variants”; *3*: “query the same variant in 50 different VCF files with 100,000 variants”

**Table 5:** Quantitative results for the three queries performed during the iDash competition

Query	1a	2a	3a
Data preparation (s)	0.04	0.4	20.2
Size of VCF file (Mbytes)	0.3	4.72	235.9
Importation (s)	0.12	1.89	94.5
PIR query generation (s)	0.04	0.05	0.67
Sending query (s)	4.77	5.03	62.9
PIR reply generation (s)	0.29	4.27	53.4
Sending reply (s)	0.53	5.19	64.9
Reply extraction (s)	0.34	4.41	55.1
**Round-trip-time** (s)	**5.5** ±**.02**	**13.3** ±**.1**	**128.1** ±**.1**
Query	1b	2b	3b
Data preparation (s)	0.037	0.36	19.2
Size of VCF file (Mbytes)	0.06	0.79	39.5
Importation (s)	0.002	0.03	1.4
PIR query generation (s)	0.008	0.016	0.2
Sending query (s)	0.736	1.78	22.3
PIR reply generation (s)	0.008	0.1	1.27
Sending reply (s)	0.31	1.15	14.5
Reply extraction (s)	0.05	0.16	2.05
**Round-trip-time** (s)	**1.07** ±**.01**	**2.95** ±**.004**	**37** ±**.5**

The data in boldface represents the round trip time, which provides an overall measure of the efficiency of our solution in the different setups

For query number *1*, in scenario *a.*, we opted for a setting with a sparser file and almost no aggregation in order to ensure that we did not retrieve more than 20 variants at a time. For the *r* *o* *w*_*s* *i* *z* *e* we chose 6, which is enough to homogenize the size of each entry. Conversely, for scenario *b.*, we chose a very compact file structure with a high number of collisions in each entry, *r* *o* *w*_*s* *i* *z* *e*=672. In this case, we no longer need to worry about obtaining extra information, as the *subtraction step* ensures that the querier obtains only access to the variants he queried.

Following an analogous reasoning, for query number *2*, we decided on 131,072 entries and a *r* *o* *w*_*s* *i* *z* *e* of 6 for scenario *a.*, and on 512 entries and a *r* *o* *w*_*s* *i* *z* *e* of 256 for scenario *b.*

Finally, query number *3* has the exact same settings as the previous query, because the number of variants in each of the 50 VCF files is the same.

To evaluate the results, we focus on the same three key elements as before: response time, storage complexity and communication cost, see Table 5.

For the first two queries, our solution provides a short execution time, no matter which scenario. We are able to query 4 variants in a VCF file with 10,000 entries in respectively 5.5 or 1.07 s, which means 1.4 or 0.3 s per variant; and 4 variants in a VCF file with 100,000 entries in, respectively, 13.3 or 2.9 s, which means 3.3 or 0.7 s per variant. The difference between the two searching methods is due to the fact that, by hiding the unnecessary variants in *b.*, we have the freedom to fully optimize the system parameters, thus obtaining a better RTT.

The last query shows that response time increases linearly with the number of variants or files queried. In fact, for each variant search in a specific VCF, we have to execute an independent PIR request each time.

## Discussion

In this section, we analyze the results reported in Tables 3 and 5 and discuss the pros and cons of our solution. We also unveil some alternative strategies for potentially addressing some of the limitations that our system can incur.

From the results of the previous section, we are confident that our solution enables the execution of a query in a short amount of time and is scalable no matter what the number of elements in the file are. Furthermore, under the right parameters, e.g, Setup *default*, and under the same encoding strategy, our solution is faster than downloading the entire database (approximately 3.5 seconds with a five million VCF file) and provides stronger security and privacy.

### Trade-offs

As previously mentioned in the Parametrization subsection, two of the most prominent and distinctive factors that affect our solution are the size of the hash for each variant, *data_hash_size*, and the number of entries in the encoded VCF file, correlated with the *bits for mapping*=*x*. Both of these parameters greatly influence the performance of our algorithm by reducing/increasing the transmission time of the VCF and of the PIR query/reply. For instance, as seen in Table 3, having a small *data_hash_size* and a compact file structure, with fewer entries and more collisions, reduces transmission time for the VCF file and the PIR query and decreases the amount of homomorphic operations performed on the server side. But, it also expands the PIR reply. Without the *subtraction step*, in addition to the answer to a query, other data are also retrieved.

Some other variables influence performance in exchange for a stronger or weaker security and privacy. The first one worth noting is the *row_size*. Recall that this parameter enforces a minimum number of elements per entry, regardless of the number of variants of a given individual. We could remove this restriction or place it below the minimum of *ceil*(5,000,000/*num_entries*), like we did for the iDash Challenge. But this would mean that the size of each entry and of the overall file would depend on the number of collisions. This would greatly improve the performance of our protocol, as seen in Table 5. But in exchange, the server could much more easily infer the actual number of variants a client has by looking at other VCF files, thus severely degrading privacy.

Therefore, if we want to achieve maximum privacy, we must ensure that all files are sufficiently large and homogeneous to hide the average maximum amount of collisions that could occur, for example, with five million variants.

We can also mention the encryption techniques and size of the symmetric key as variables that are engaged in this trade-off. Having symmetric encyption, AES-CTR, slightly decreases performance but ensures data confidentiality. Finally, opting for a larger or smaller polynomial for HE dictates the security level, the amount of aggregation we can do, as well as the size of the PIR query and reply (see Setup *security* in Table 3).

### Features

Our strategy features the following properties:

- **Optimal privacy**: By hashing and padding each file with dummy data, we ensure that no matter how many variants, with a maximum of five million, all files are indistinguishable. Therefore, we can hide the length of each file and the length of its elements. Using PIR hides access patterns and provides inalienability when querying the same variant twice. We can weaken the privacy level and in exchange achieve better performance.
- **Confidentiality**: We symmetrically encrypt the data using AES-CTR256.
- **Good security scalability**: As we rely on lattice-based cryptography to perform PIR, we are able to increase security without much performance overhead, e.g., increasing security by a factor of two only decreases performance by a factor of two.
- **Low storage-complexity**: By using hashing, we significantly reduce the size of the VCF files. Hence, if for example we store 48 bits (6 bytes) of the hash, every VCF file will be around 30 Mbytes (5,000,000×6). To maintain privacy, we need to pad enough dummy data, thus hiding the number of variants.
- **Low querying-time**: This solution yields a fast querying time and is extremely scalable. Under the stronger padding scheme, querying a file with 1 variant takes the same time as querying a file with five million variants.
- **Minimization of delivered data**: We propose a way to reveal only the queried variants to the client. This is accomplished by means of the additional *subtraction step* executed after revealing the PIR reply.
- **Generality**: We consider all known variants, not only single-nucleotide polymorphisms (SNPs). Furthermore, our solution can easily store a different encoding instead of its hash, possibly enabling other kinds of operations on the genomic data.

### Limitations

There are a few limitations that we can identify in our strategy. The first is the error rate associated with our hashing scheme. In the next section, we propose a way to mitigate this problem, and we still provide a fast querying time. Second, if the number of VCF files in the database is relatively large, then we can no longer store pre-imported data into RAM. This can be solved by storing pre-imported data into a disk, but will result in a slow down. As reply generation is more rapid than sending the reply, the overhead due to the disk reading will not affect RTT, except for very slow disks. Finally, our default strategy still suffers from some scalability issues, especially if multiple variants or files are queried - *O(n)* complexity, with *n* being the number of variants or files queried.

### Other possible strategies

We now list a couple of other alternative strategies and mechanisms for addressing some of the previously listed shortcomings of our protocol. These ideas have both pros and cons, some of which we detail in the following paragraphs.

The first idea is to store the encoding of a variant instead of part of its hash. This would remove the risk of having false positives and enable the possible execution of other operations on the data.

**Encoding exaple** uses information on variant type [op-2bits] (*insertion, deletion, single polymorphism, substitution*), chromosome [chr-5bits], position [pos-28bits], reference [ref-2bits/base] and alternate [alt-2bits/base] alleles.

$$\begin{array}{@{}rcl@{}} \begin{array}{cc} \mathrm{INSERTION/SNP:}\\ \mathbf{op (01)\|chr\|pos\|alt}\\ \mathrm{DELETION:}\\ \mathbf{op (11)\|chr\|pos\|ref length}\\ \mathrm{SUBSTITUTION:}\\ \mathbf{op (11)\|chr\|pos\|ref \;length\|alt}\\ \mathrm{EXAMPLE:}\\ \mathrm{chr: 1; position: 160999478; reference: A; alternate: G}\\ 01 00001 1001100110001010100000110110 01 10\\ \end{array} \end{array} $$

However, in addition to the normal padding, we would also need to hide each variant’s length by means of another padding. With this particular encoding, variant size varies from a SNP (37 bits, see example above) to an insertion/deletion (variable, e.g., 81 bits). Thus, padding each element to the maximum encoding length in the database would increase the database size by a factor of 2 with the proposed example. Larger databases significantly degrade performance (see Table 6) because they increase importation time and PIR reply-generation/reply-sending.

**Table 6:** Quantitative results relative to our alternative encoding strategy. We use the same settings as Setup *default* but with *d* *a* *t* *a*_*h* *a* *s* *h*_*s* *i* *z* *e*=81

Setup	Encoded
Data preparation (s)	22.3
Size of VCF file (Mbytes)	58.6
Importation (s)	1.02
PIR query generation (s)	0.03
Sending query (s)	1.37
PIR reply generation (s)	0.62
Sending reply (s)	1.68
Reply extraction (s)	0.66
**Round-trip-time (RTT)** (s)	**3.06** ±**.004**

The data in boldface represents the round trip time, which provides an overall measure of the efficiency of our solution in the different setups

The second idea, which we briefly discussed in the previous subsection, is to have a much lengthier database with no collisions, thus removing the need to perform the *subtraction step*. This would also enable a much faster AND operation by means of a point-value polynomial representation. This way, we could batch several queried elements into one single PIR query; instead of one query per variant, and then search, in the reply, for the value corresponding to the addition of the variants. This approach, however, would require a huge database and much dummy data, thus severely degrading the performance of the PIR protocol. One naive way to avoid collisions could be to separate each variant into a collection of SNPs to be encoded (37 bits) and directly place them in a database with 2^37^ entries. Cuckoo hashing [26] offers a smarter alternative, by replicating the database and using two hashes/positions for each single element. However, for our algorithm to work, we require one of the two positions to remain empty, something that cannot be efficiently done with the standard greedy insertion algorithm.

**Fig. 7:**
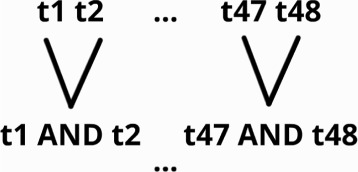
FHE-only scheme. The response is 1 if an element matches, 0 if not. Unfortunately this kind of multiplication is very time consuming

Finally, the third alternative would be to not use PIR and instead employ a fully-homomorphic encryption (FHE) scheme. For this protocol, the database would contain encrypted multi-nucleotide variants unordered. To perform a query, we would use FHE to test the equality of each database element to, for example, a 48-bit homomorphically encrypted hash as shown in Fig. 7. We would do this by sending 48 queries that correspond to the 48 bits of the encrypted variant’s hash we want to verify. Then, because the noise increases significantly with multiplicative depth, we would apply a binary tree on each entry of the database.

**Notation:** *a*_*i*_ is the *i* ^*th*^ bit we send; *b*_*i*_ is the *i* ^*th*^ bit of an element in the database; *t*_*i*_ represents the equality test of the bits *i*. We can do this by working modulo 2 with: 1+(*a*_*i*_+*b*_*i*_).

We list some of the pros and cons for the FHE-only alternative strategy.

**Pros:**

- Small database size (small-sized padding);
- The size of the reply does not depend on the variants’ representation and is a simple binary response: 0 or 1;
- Easy AND operations.

**Cons:**

- Too much time to generate the response.

## Conclusion

We have described a new efficient strategy that uses private information retrieval to search genomic variants on a cloud database. This scheme makes use of a new enhanced PIR protocol that we adapt to fit our purpose. All the security and privacy requirements were met by introducing certain modifications in the PIR protocol, such as the need to homogenize each client’s VCF file and the symmetric encryption of the data to guarantee confidentiality and privacy. We have also listed some other alternative mechanisms that can be useful, depending on the setting, e.g., sparse database and cuckoo hashing to reduce AND complexity, or encoding to enable other operations. Finally, results show that, although not as effective as a simple search through an unencrypted database, this strategy exhibits a good performance and could be realistically deployed, for example, in clinics or hospitals. Future work includes finding a way to make AND operations scalable, probably by means of cuckoo hashing.

## Abbreviations

HE: Homomorphic encryption
PIR: Private information retrieval
RTT: Round-trip-time
VCF: Variant call format
